# A Review of Carbon Nanomaterials’ Synthesis via the Chemical Vapor Deposition (CVD) Method

**DOI:** 10.3390/ma11050822

**Published:** 2018-05-17

**Authors:** Yehia M. Manawi, Ihsanullah, Ayman Samara, Tareq Al-Ansari, Muataz A. Atieh

**Affiliations:** 1 Qatar Environment and Energy Research Institute (QEERI), Qatar Foundation, P.O. Box 5825, Doha, Qatar; ymanawi@qf.org.qa (Y.M.M.); asamara@hbku.edu.qa (A.S.); 2 College of Science and Engineering, Hamad Bin Khalifa University (HBKU), Qatar Foundation, P.O. Box 5825, Doha, Qatar; 3 Center for Environment & Water (CEW), Research Institute, King Fahd University of Petroleum and Minerals (KFUPM), Dhahran 31261, Saudi Arabia; 4 Division of Sustainable Development, College of Science and Engineering, Hamad Bin Khalifa University(HBKU), Qatar Foundation, P.O. Box 5825, Doha, Qatar; talansari@hbku.edu.qa

**Keywords:** chemical vapor deposition, fullerene, carbon nanotubes, carbon nanofibers, graphene, carbide-derived carbon, carbon nano-onion, MXenes

## Abstract

Carbon nanomaterials have been extensively used in many applications owing to their unique thermal, electrical and mechanical properties. One of the prime challenges is the production of these nanomaterials on a large scale. This review paper summarizes the synthesis of various carbon nanomaterials via the chemical vapor deposition (CVD) method. These carbon nanomaterials include fullerenes, carbon nanotubes (CNTs), carbon nanofibers (CNFs), graphene, carbide-derived carbon (CDC), carbon nano-onion (CNO) and MXenes. Furthermore, current challenges in the synthesis and application of these nanomaterials are highlighted with suggested areas for future research.

## 1. Introduction

Materials can be referred to as nano-scaled when their size range from approximately 1 nm to 100 nm [1]. Nanomaterials have gained considerable attention in various fields owing to their tremendous properties [2,3]. Carbon, one of the most abundant materials found on earth, can be found in nature in its elemental form as graphite, diamond and coal [4,5]. The carbon nanomaterials with their excellent properties are ideal candidates for advanced applications in the area of electronics, membranes, wastewater treatment, batteries, capacitors, heterogeneous catalysis, as well as biological and medical sciences [6,7,8,9,10,11,12,13,14,15,16,17].

**Figure 1:**
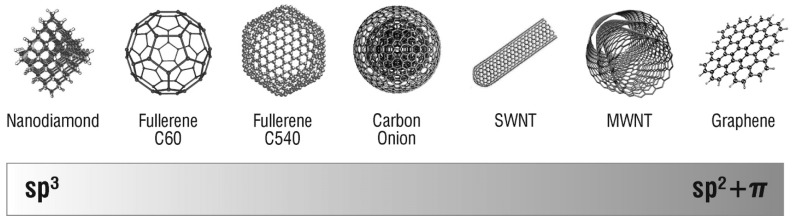
Hybridization states of carbon-based nanomaterials. Reproduced with permission from [4]. Copyright (2008) American Chemical Society.

It is well known that the morphologies, sizes and phases of nanomaterials have great influence on their properties and potential applications [18]. Therefore, the synthesis of nanostructured materials with desired properties has recently received much attention [19,20,21]. Carbon can be found in several different hybridization states, each having unique properties as shown in Figure 1 [4]. In fact, the electrical, thermal, mechanical and chemical properties of the different allotrope forms are directly correlated to their hybridization state and structure, opening up the possibility to use the same material for a wide range of applications [4].

Various techniques have been reported in the literature for the synthesis of 0D, 1D, 2D, and 3D carbon nanomaterials. The most common techniques are laser ablation [22,23,24,25], arc-discharge [25,26] and chemical vapor deposition (CVD) [27,28]. CVD is the most commonly employed thin-film deposition technique used to synthesize nanomaterials. Therefore, this review paper will be mainly focused on the synthesis of novel materials via the CVD technique.

This review paper summarizes the synthesis of carbon nanomaterials such as fullerenes, carbon nanotubes (CNTs), carbon nanofibers (CNFs), graphene, carbide-derived carbon (CDC), carbon nano-onion (CNO) and MXene via the CVD method.

## 2. Synthesis of Carbon Nanomaterials 

### 2.1. Fullerenes

**Figure 2:**
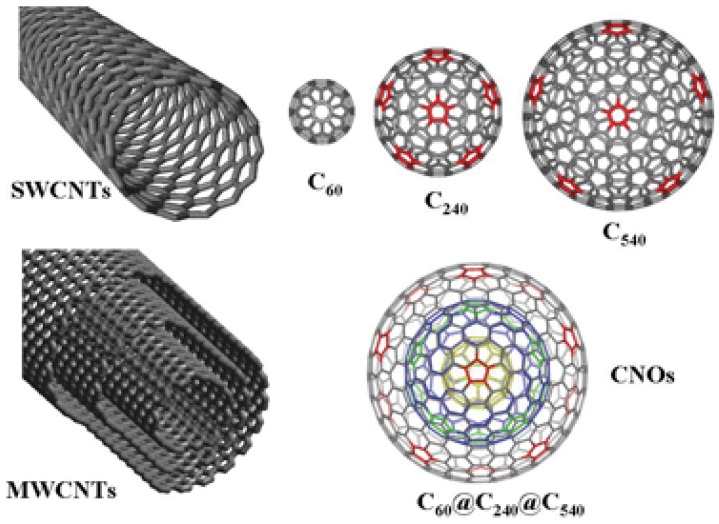
Types of fullerenes. Reproduced with permission from [33]. Copyright (2010) Royal Society of Chemistry.

Fullerene is an allotrope of carbon which has a hollow structure that takes the shape of the sphere, tube, ellipsoid in addition to some other structures [29]. By definition, a fullerene is a closed cage molecule containing only hexagonal and pentagonal faces [30]. Spherical fullerenes look like balls and that’s why they are sometimes referred to as Bucky-balls. On the other hand, bucky-tube is the name used to denote cylindrically shaped fullerene or carbon nanotubes. Structure-wise, all fullerenes are similar to graphite which consists of arranged graphene sheets (composed of linked hexagonal rings) [29]. Additionally, fullerene could also consist of pentagonal or heptagonal rings. Buckminsterfullerene (C_60_), was the first Fullerene to be discovered in 1985 by Richard Smalley et al. [31]. In fact, this structure had been identified by Sumio Iijima in 1980 using an electron microscope image [32]. The identified structure was the core of the bucky onion or CNOs [32]. Fullerene has widened the family of carbon allotropes which includes CNTs, graphene, charcoal, soot, CNOs, etc. (as shown in Figure 2).

#### 2.1.1. Synthesis of Fullerenes via Chemical Vapor Deposition (CVD)

Fullerenes can be synthesized using several techniques; the most commonly used ones are laser ablation [25], arc vaporization of graphite [25], and CVD methods [34,35]. Fullerenes are traditionally produced by the CVD method, combustion processes and arc-discharge vaporization of graphite. However, these methods are not very efficient, and continued refinements and improvements in this area are needed. Therefore, developing new, efficient methods for the synthesis of fullerene with high yields and purity is still a challenge for researchers. The CVD method for the production of fullerenes, however, has the advantage that lower temperature is required as compared to laser vaporization and arc discharge. Kleckley et al. [34] presented two methods for the synthesis of fullerene that are hot-filament CVD and microwave-enhanced CVD methods.

##### Hot-Filament CVD

**Figure 3:**
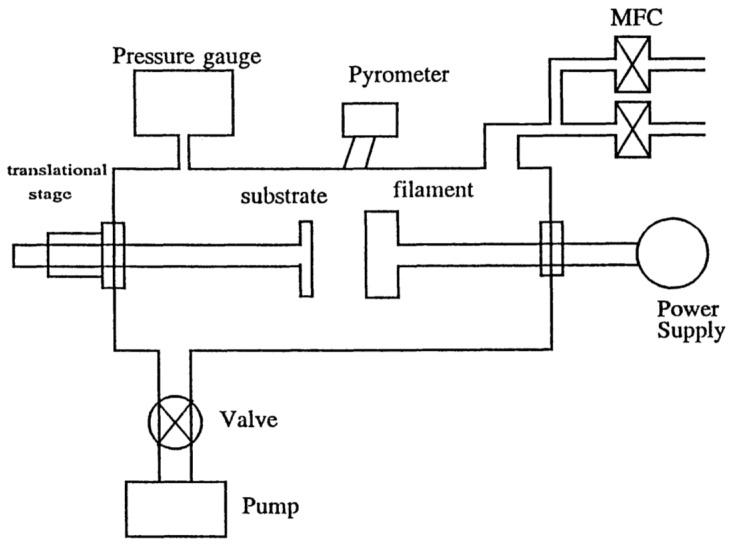
Schematic diagram of the hot-filament chemical vapor deposition (CVD) chamber. Reproduced with permission from [34]. Copyright (1997) American Chemical Society.

A schematic diagram of the hot-filament CVD is shown in Figure 3 [34]. The chamber is constructed of stainless steel with a filament made of tungsten wire. The filament hangs vertically, with the lower terminal attached to a braided copper wire and the upper terminal fixed. A stainless steel substrate holder is used for thin-film deposition. The filament temperatures are typically from 2000 °C to 2200 °C and the filament currents are between 50 A and 60 A. The typical substrate temperatures are between 950 °C and 1000 °C for the growth of CVD diamond-thin films. The feed gases are 99.999% pure hydrogen and 99.8% pure methane. The pressure of the chamber is controlled in the range of 30–100 Torr.

##### Microwave-Enhanced CVD

**Figure 4:**
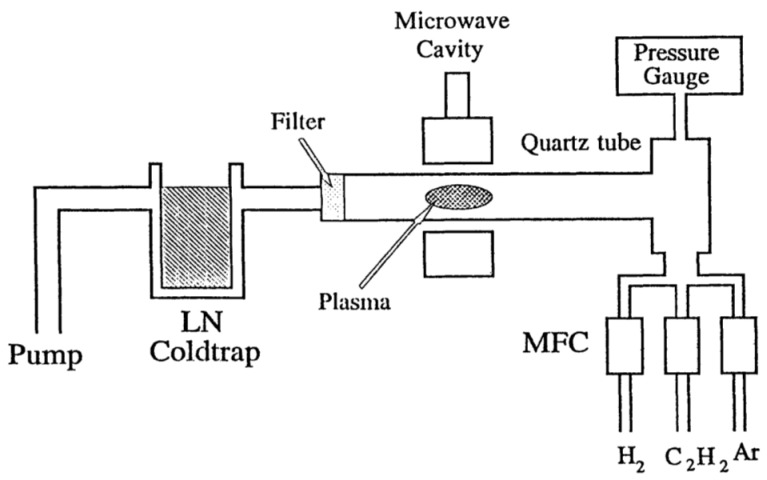
Schematic diagram of the microwave-enhanced CVD chamber. Reproduced with permission from [34]. Copyright (1997) American Chemical Society.

Microwave CVD apparatus is presented in Figure 4 [34]. A quartz tube was used as a reaction chamber. A 100 W 2.45 GHz generator was used as the excitation source, which was connected to an Evenson-type cavity. The feed gases we used included Ar, H_2_ and C_2_H_2_. Typical pressures were between 1 Torr and 10 Torr. It was found that at *p* > 25 Torr, a conducting film was building up inside the quartz tube, which limited the deposition process to only a few minutes. At lower pressure, it was observed that the plasma region was much more extended, and it resembled a glow discharge plasma. At *p* < 10 Torr, a yellowish film was deposited on the inside wall of the quartz tube. The film color changed to dark brown after being exposed to the plasma for 30 min.

### 2.2. Carbon Nanotubes (CNTs)

**Figure 5:**
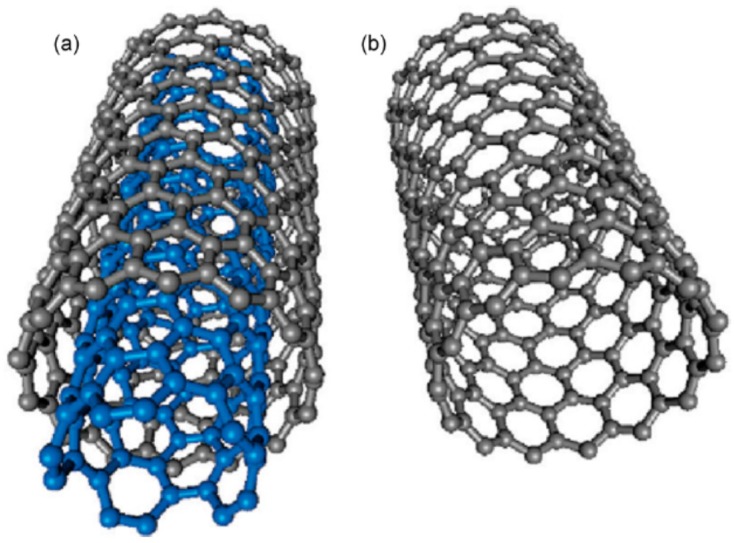
Structure representation of (**a**) multi-walled carbon nanotubes (MWCNTs); (**b**) single-walled carbon nanotubes (SWCNTs). Reproduced with permission from [37]. Copyright (2009) American Chemical Society.

**Figure 6:**
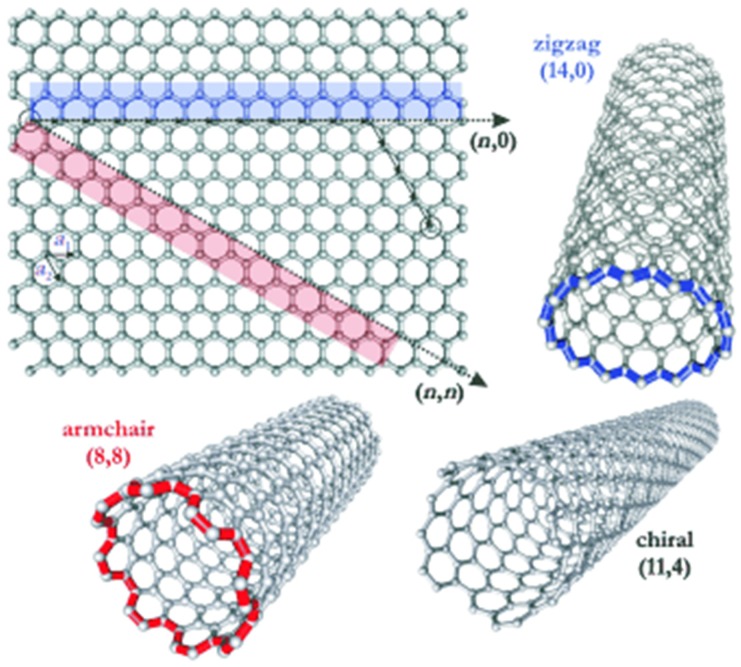
Three different structures of carbon nanotubes (CNTs). Reproduced with permission from [38]. Copyright (2005) Wiley Publishers.

Carbon exist in many molecular forms, known as allotropes of carbon. Carbon nanotubes (CNTs) are allotropes of carbon that are composed of cylindrical graphite sheets rolled up in a tube-like structure [36]. CNTs that are composed of a single graphene sheet are termed as single-walled carbon nanotubes (SWCNTs). Conversely, multilayers of graphene sheets are known as multi-walled carbon nanotubes (MWCNTs). The multi-walled nanotubes (MWCNTs) and single-walled carbon nanotubes (SWCNTs) are depicted in Figure 5 [37]. Figure 6 depicts several SWCNT structures based on the way graphene sheets are rolled [38].

Chiral indices (*n*,*m*) is an index used to identify the structure of the CNTs according to the orientation of the tube axis with respect to the hexagonal lattice. The origin of the zigzag (*n* = 0) and armchair (*n* = *m*) nanotubes’ structure was adopted from the geometric arrangements of carbon atoms at the cylinder’s seam. Nanotube structures with a characteristic two enantiomers with right and hand side helicity (*n* ≠ *m*) are called chiral [38].

In 1991, Sumio Ijima at the NEC Laboratory in Tsukuba, Japan, discovered CNTs using the arc-discharge technique. These CNTs were then characterized using a high-resolution transmission electron microscope (HRTEM) [39]. Chemical bonding in nanotubes is composed entirely of sp^2^ bonds, which are stronger than the sp^3^ bonds found in alkanes and provide nanotubes with unique strength [40]. Interestingly, CNTs have much higher length-to-diameter ratio than other existing materials (i.e., up to 132,000,000:1) [40].

Due to their unique hexagonal structure, CNTs possess some extraordinary properties (electrical, mechanical and thermal) which make them versatile in many applications in various fields [41,42]. Being part of the fullerene family, the name of CNTs was derived from the long hollow hexagonal cylindrical structure which has one-atom thick walls made from carbon sheets referred to as “graphene”. The respective sheets are then rolled at specific chiral angles. Eventually, the properties of the rolled CNTs are decided by the radius and the rolling angle. Normally, CNT cylinders are topped with a fullerene-type molecule [43]. In fact, there are two types of CNTs i.e., single-walled nanotubes (SWCNTs) and multi-walled nanotubes (MWCNTs) as shown in Figure 5. Each of these two types of CNTs aligns themselves as ropes while being attracted to each other through van der Waals forces [44].

A single graphene sheet has rolled up to form a cylindrical tube (SWCNT) with a diameter ranging between 0.4 nm and 3 nm [45]. On the other hand, MWCNTs can be defined as concentric cylinders made from graphene sheets with diameters up to 100 nm [46]. The structure of SWNTs can be determined by wrapping the constituting graphene sheet into a cylinder. As shown in Figure 6, the way this graphene sheet is wrapped is expressed by the indices *n* and *m* (or *n*,*m*). In this way, the indices *n* and *m* signify the number of unit vectors along the two directions in the honeycomb crystal lattice of graphene. When index *m* = 0, the structure of the nanotube is referred to zigzag; however, when *n* = *m*, the structure of the nanotube is called armchair. Otherwise, the nanotubes’ structure is referred to as chiral [47].

#### 2.2.1. Synthesis of CNTs

The most common methods for CNT production are CVD [47,48,49], laser ablation [24] and electric arc discharge [50,51]. However, CVD has proven itself as a favored method for the mass production of CNTs [52,53]. Various CVD techniques employed for the production of CNTs include hot-wire (HWCVD), hot-filament (HFCVD), microwave plasma-enhanced (MWCVD), oxygen-assisted, aerosol-assisted (ACVD) and liquid-injection (LICVD).

##### Synthesis of CNTs via CVD

CVD is the most commonly used thin-film deposition technique used to synthesize CNTs. CVD is a different method from the other CNT synthesis methods. The CVD method for the production of CNTs has the advantages of high-yield of nanotubes and a lower temperature requirement (550–1000 °C) that makes the process both cheaper and more accessible for lab applications. Furthermore, the CVD method allows control over the morphology and structure of the CNTs produced and the growth of aligned nanotubes in a desired direction is possible. However, the CVD method has the disadvantage that the nanotubes are more structurally defective than those produced by laser evaporation or the arc discharge method. Both arc discharge and laser vaporization are considered as short-reaction time (micro to milli-seconds) and high-temperature processes (above 3000 K) [50]. On the other hand, catalytic CVD is a long-time reaction (minutes to hours) requiring intermediate temperatures (700–1473 K).

The CVD technique for mass production of CNTs employs hydrocarbon or other carbon-bearing precursors in the presence of a catalyst, and CNTs are deposited on to a substrate. The typical temperature in a CVD process is normally below 1200 °C. This process has the advantage of producing CNTs with the desired structure by controlling their alignment, length, wall number and diameter. Generally, CVD is considered to be the low-cost process for the production of CNTs.

**Figure 7:**
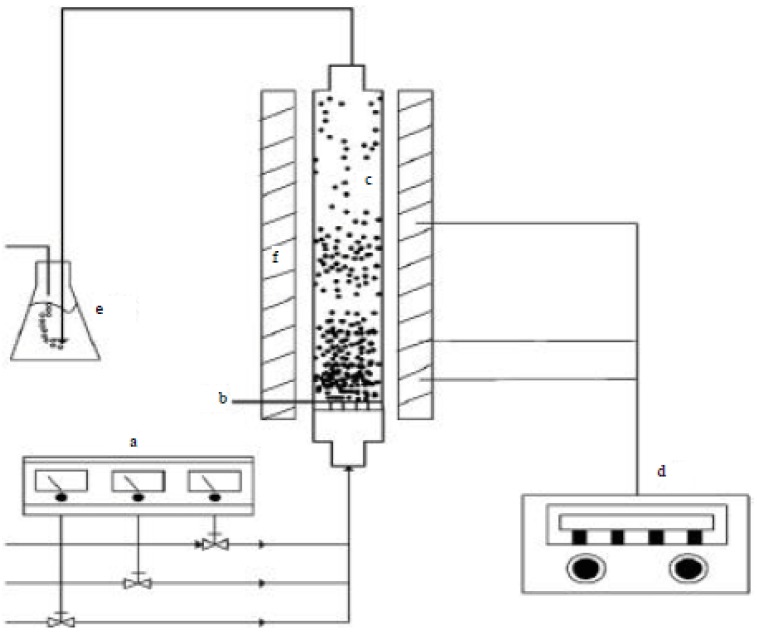
Schematic diagram of the fluidized-bed reactor for growing CNTs. Reproduced with permission from [61]. Copyright (2014) Academic Journals Inc.

The most common way for large-scale production of CNTs is to use the fluidized-bed process [54,55,56,57,58,59,60]. Figure 7 shows a schematic of a typical fluidized-bed reactor, where Figure 7a–f represents the mass-flow controller, gas distributor, fluidized-bed reactor, temperature controller, cold-trap system and furnace, respectively [61].

The basic steps involve the dissociation of hydrocarbon molecules, atomic carbon saturation in the catalyst nanoparticles, and carbon precipitation from catalyst in order to form CNTs [62]. The role of the catalyst is very critical in the mass production of CNTs [63]. A number of transition metals e.g., Fe, Mo, Co, Ni, ferrocene and iron pentacarbonyl can be used as a catalyst; however, iron is the most extensively used catalyst for the synthesis of CNTs [52,59].

CNTs produced by this method do not grow on a patterned or conventional substrate [56]. High-quality MWCNTs and SWCNTs can be grown directly in bulk as a raw material or into the substrate. No purification is required in this method unless the catalyst metal needs to be removed [22]. This method requires growing CNTs by decomposing organic gas on top of a substrate covered with metal catalyst particles [22]. There are many methods used to fabricate CNTs using CVD; the most common methods are plasma-enhanced CVD, catalytic pyrolysis of hydration, and thermal CVD [64].

**Figure 8:**
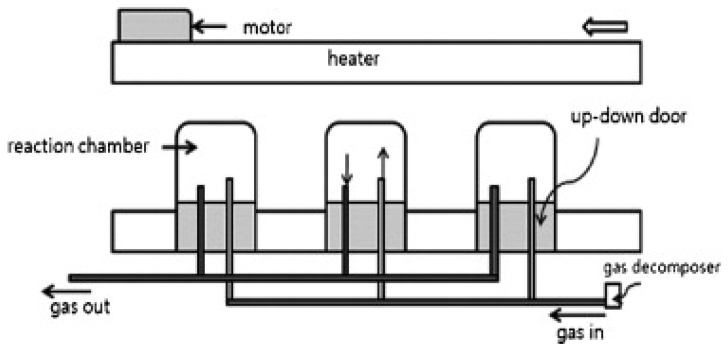
Schematic of fluidized-bed reactor using a floating catalyst. Reproduced with permission from [65]. Copyright (2009) Hanyang University.

Figure 8 shows a schematic of a CVD reactor having catalyst-containing ceramic boats within the reactor. The gas mixture interacts directly with the catalyst in the reaction chamber and results in the continuous production of CNTs [47,65].

**Figure 9:**
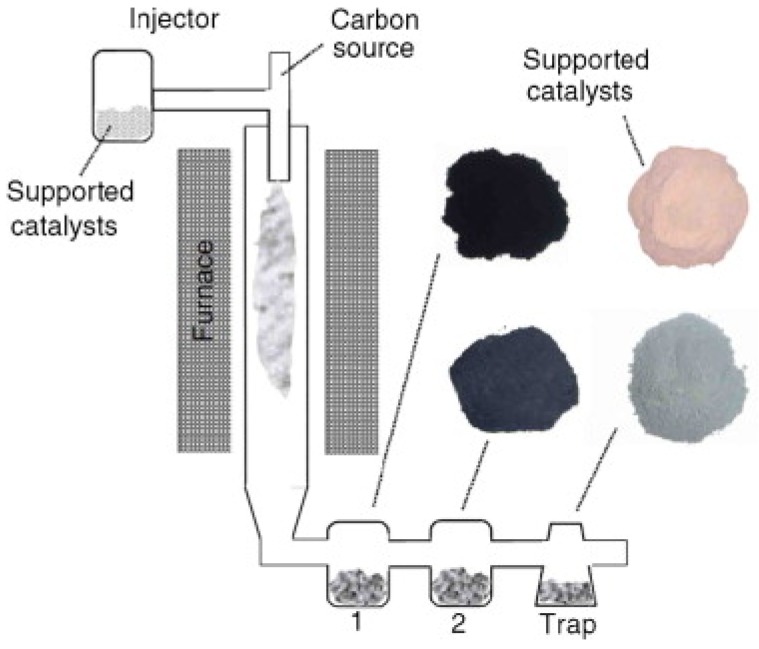
Schematic of vertical floating quartz tube reactor. Reproduced with permission from [66]. Copyright (2007) Elsevier B.V.

Figure 9 show a modified vertical floating-quartz tube reactor with alumina-supported Fe/Mo catalyst powder. The catalyst powder was injected into the reactor using argon as a carrier gas, while methane gas was used as a carbon source. The carbon product formed was carried away by the carrier gas and stored in the collector [66].

**Figure 10:**
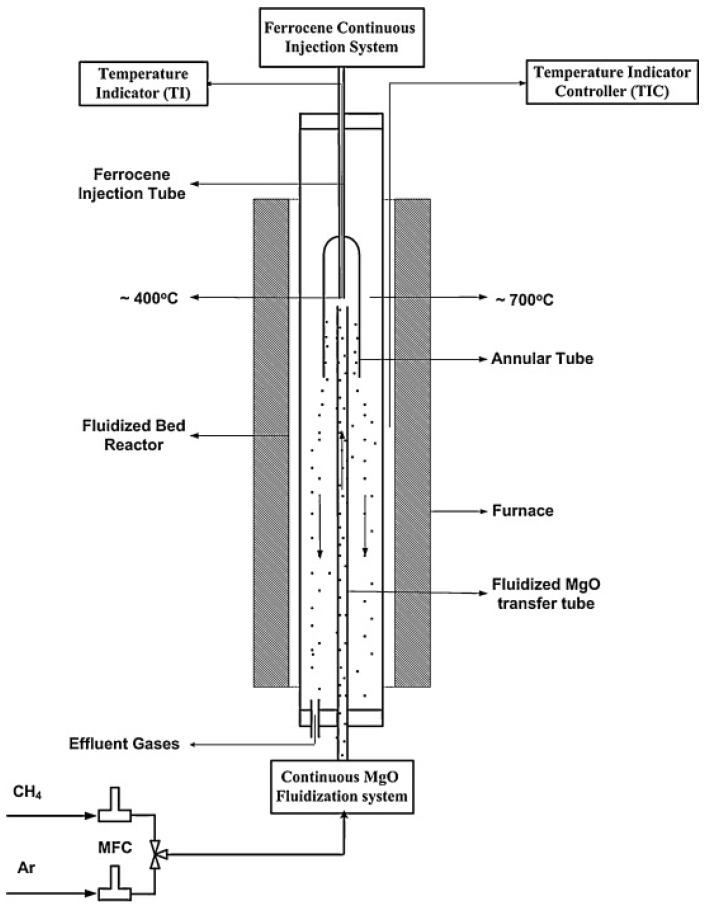
Schematic diagram of the continuous fluidized-bed system used for CNT synthesis. Reproduced with permission from [67]. Copyright (2010) Elsevier B.V.

Figure 10 shows another modified fluidized-bed reactor, with a blend of fluidization method and floating catalyst. The ferrocene is injected at the top of the reactor and reacts with the iron floating Fe/MgO catalyst. In this way single-wall and double-wall CNTs are grown on the surface of the Fe/MgO catalyst [67].

Lehman et al. [68] reported the production of CNTs by the hot-wire CVD method (HWCVD). The HWCVD was performed in a quartz tube reactor enclosed in a clamshell furnace. CNTs were grown on a lithium niobate (LiNbO_3_) pyroelectric detector with a nickel film as the catalyst. CNTs were produced at 600 °C and at 150 Torr in 1:5 CH_4_:Ar.

Makris et al. [69] produced CNTs by hot-filament chemical vapour deposition (HFCVD) methods using nickel catalysts. A DC current (with electrical power of approximately 1.5 kW) was fed into eight 0.8 mm-thick × 100 mm-long straight Ta filaments inside the HFCVD reactor chamber. The temperature was maintained at approximately 1800 °C. CNTs were grown on silicon native oxide and on a substrate with a SiO_2_ coating between the Si substrate and Ni film using the gas mixtures of H_2_ and CH_4_ as gas precursors.

Microwave plasma-enhanced CVD (MWCVD) can also be used for the production of CNTs. Choi et al. [70] reported the growth of carbon nanotubes on Ni-coated Si substrates by microwave plasma-enhanced CVD at low temperature. A mixture of H_2_ and CH_4_ gases was used at temperatures ranging from 520–700 °C. It was observed that the carbon nanotubes were curly at 520 °C, whereas at temperature above 600 °C, the nanotubes were straight. Similarly, Watanabe et al. [71] synthesized Boron-doped carbon nanotubes by the MWCVD method. Methane and trimethyl-borate gas were used as source materials. Iron was used as a catalyst. Hydrogen gas was used to induce plasma. The CNT growth temperature was 700 °C, the ratio of methane gas to hydrogen gas was 1:4, the catalyst thickness was 3 mg, the chamber pressure was 15 Torr, and the microwave power was 400 W.

Byon et al. [72] synthesized high-purity single-walled carbon nanotubes (SWCNTs) from small diameters of cobalt (Co) nanoparticles by using oxygen-assisted CVD. The Co catalyst nanoparticles were employed for the growth of pure SWNTs in an oxygen-assisted CVD condition. The temperature was raised to 900 °C in the quartz tube and different gases i.e., CH_4_, H_2_ and C_2_H_4_, were introduced into the chamber. The small quantity of oxygen flow at high temperature removed amorphous carbons and eliminated unreacted catalysts from the substrate. It was revealed that a high purity of SWNTs without severe defects can be obtained in the oxygen-assisted CVD condition.

Szymanski et al. [73] reported the synthesis of carbon nanotubes in a thermal plasma reactor at atmospheric pressure. The substrate for the synthesis was made of stainless steel and the substrate temperature (1000–1300 K) was controlled by the pyrometer. A mixture of ethylene, hydrogen, nitrogen and argon was introduced orthogonally to the plasma jet, while nitrogen and argon were supplied axially to the microwave plasma nozzle. The synthesis of carbon nanotubes took place in the chamber on a metal strip (stainless steel) prepared by depositing a catalyst layer on the strip surface.

Meysami et al. [74,75,76] reported the large-scale production of CNTs by using the aerosol-assisted CVD method (ACVD). CNTs were synthesized using an ACVD system consisting of a piezo-driven aerosol generator and a quartz tube placed inside a 60-cm-long horizontal tube reactor. Argon was fed to the reactor prior to synthesis of the CNTs. For the growth of CNTs, the aerosol generator was filled with a precursor solution containing 5 wt % ferrocene and 95 wt % toluene and connected to the quartz tube. The aerosol generator chamber was flushed with argon at room temperature prior to the growth of CNTs. When the reactor reached 800 °C, the aerosol generator was turned on and switched off After 15 min, it was cooled under argon flow, and the substrates were carefully removed for characterization. The potential for up-scaling the production of CNTs via the ACVD method has been reported in a later study by the same researchers. Some other researchers have also reported the production of high-purity vertically aligned films of multi-wall carbon nanotubes via an aerosol-assisted CVD method [77,78].

Liquid injection chemical vapor deposition (LICVD) is another useful technique for the large-scale production of CNTs. The injection CVD method involves pumping or spraying a metallocene–hydrocarbon solution into a suitable furnace. This method has many advantages including excellent control of the catalyst-to-carbon ratio, the nanotube length diameter, and alignment. The injection-CVD method does not require a catalyst synthesis step, since the catalytic particles are generated in situ continuously throughout the entire growth cycle. This offers the possibility of scaling up the method for continuous or semi-continuous production [79]. Singh et al. [80] reported the production of high-purity, aligned multi-walled carbon nanotubes by the CVD injection method. The CNT films were grown on quartz substrates by injecting a solution of ferrocene in toluene in a temperature range of 550–940 °C. The nanotubes were collected from quartz substrates placed in the center of the hot zone.

Horváth et al. [81] produced MWCNTs by the spray-pyrolysis method. The effects of various hydrocarbons (benzene, toluene, xylene, cyclohexane, cyclohexanone, *n*-hexane, *n*-heptane, *n*-octane and *n*-pentane) and metallocenes (ferrocene, cobaltocene and nickelocene) were investigated for their influence on the quantity and quality of the CNTs. The maximum yield was found when xylene was used as a carbon source and a ferrocene–nickelocene catalyst mixture as a catalyst. Hayashi et al. [82] produced a free-standing single-walled carbon nanotube, which has a diameter of about 0.43 nm by the improved floating reactant method. This method combines the conventional substrate and floating catalyst methods using zeolite particles as a floating catalyst support. Table 1 lists the different catalytic methods for the production of SWCNTs, MWCNTs and double-walled carbon nanotubes (DWCNTs) by CVD and their experimental condtions.

### 2.3. Carbon Nanofibers (CNFs)

**Figure 11:**
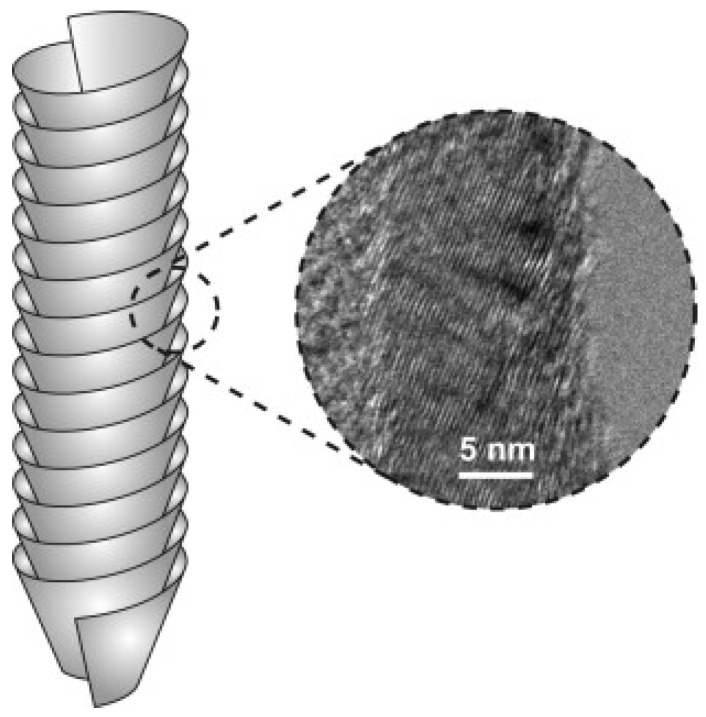
Schematic stacked-cup carbon nanofiber structure with a transmission electron microscope (TEM) image showing the inclined orientation of the stacked graphene sheets with respect to the nanofiber axis. Reproduced with permission from [118]. Copyright (2011) Elsevier B.V.

Carbon nanofibers (CNFs) are hollow-core nanofibers consisting of a single graphite layer or double graphite layers which are stacked parallel or at a specific angle from the fiber axis [109]. The stacked layers are located next to each other and have different structures including parallel, cup-stacked and bamboo-like structure [110]. As shown in Figure 11, CNFs are cylindrical nanostructures which consist of graphene layers that are arranged as stacked cones, cups or plates. When CNFs with graphene are formed into cylinders, they are referred to CNTs. CNFs have gained less attention when compared with CNTs, as CNTs have a smaller diameter, lower density and better mechanical properties (as a result of fewer microstructural defects present in CNFs) [111]. However, CNFs are considered perfect substitutes for CNTs due to their low price and availability [109]. Carbon fibers have major industrial applications, and their growth mechanism and the factors which control their structure are of strategic importance [112]. Moreover, due to their relatively cheap synthesis cost, CNFs can be studied and tested for the sake of applying acquired knowledge to the expensive CNTs [113]. The fabrication cost of MWNTs is 2–3 times higher than CNFs, while that of the SWNTs is even higher. Developments in fabrication science are expected to lower the fabrication cost even further [113]. The exceptional features of CNFs have widened their applications. CNFs are now used by scientists to fabricate composite materials with properties much better than existing materials [114]. CNFs have attracted a lot of attention due to their unusual electrical, thermal and mechanical properties. CNFs are now utilized in electrical applications due to their lower loading in order to achieve certain electrical conductivities. The properties of CNFs largely depend on their structure, which depends on the synthesis technique (catalysts, feedstock, etc.) and post-treatment techniques [115,116,117].

#### Synthesis of CNFs via CVD

**Figure 12:**
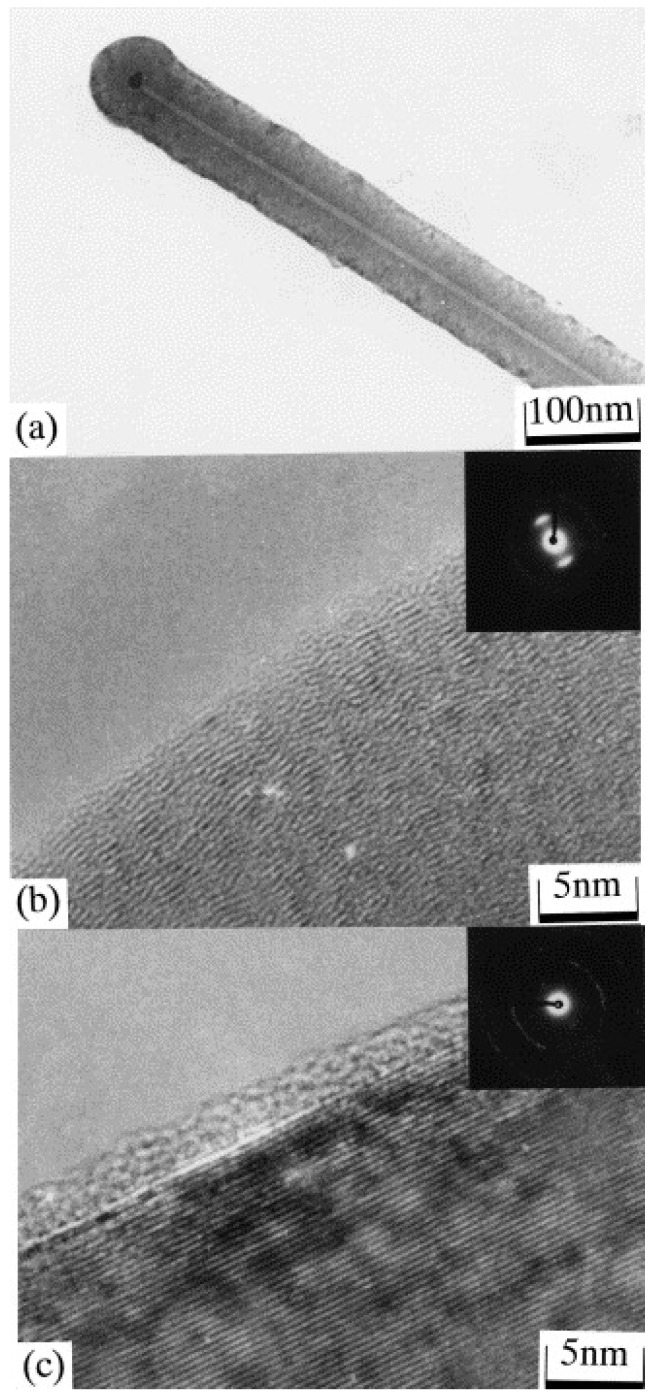
Transmission electron micrographs of (**a**) as-grown submicron vapor-grown carbon fibers (VGCFs) at low magnification; (**b**) lattice image in the external part of the as-grown fiber; and (**c**) lattice image in the external part of the graphitized fiber. Reproduced with permission from [110]. Copyright (2001) Elsevier B.V.

Large-scale production of CNFs is possible via CVD methods. CVD has the advantage of tailoring the diameter, crystallinity, and also orientation of the fiber axis through precise control of the synthesis conditions. CNFs are synthesized by the catalytic CVD of a hydrocarbon (propane, benzene, ethylene, acetylene or natural gas) or carbon monoxide over a metal surface (Ni, Fe, Co or Au) or metal alloy catalyst (such as Fe-Ni or Ni-Cu) [119,120,121]. The catalyst can be fed with the gas phase or deposited on a substrate [121,122]. The reaction takes place at a temperature range of 500–1500 °C [122]. Pyrograf® III nanofibers (Applied Sciences Inc. (ASI), Cedarville, OH, USA) are well-studied CNFs which are synthesized using a gas-phase reactor at 1100 °C. Natural gas is fed to the reactor along with ammonia, hydrogen sulfide (to activate and disperse the catalyst) and a metal catalyst (Fe(CO)_5_). The decomposition of hydrocarbon on the surface of the metal catalyst is responsible for synthesizing CNFs [123]. The reaction time is in the order of milliseconds and the synthesized CNFs have a high resistivity. The morphology and characteristics of the CNFs obtained depend on the type of catalyst, the feedstock used, and the operating conditions used [123]. Vapor-grown carbon fibers (VGCFs) are discontinuous short fibers with high electrical conductivities [124]. Transmission electron micrographs of VGCFs (synthesized by Endo et al. [110]) are shown in Figure 12.

In another approach, activated carbon (AC) is used as substrate to grow CNFs [125,126,127,128]. When AC is used, post-synthesis processing is not required because the prepared hierarchical micro–mesoporous structure of CNF/AC can be directly utilized in end-applications such as in the fabrication of fuel-cell electrodes and adsorbents for environmental remediation.

**Figure 13:**
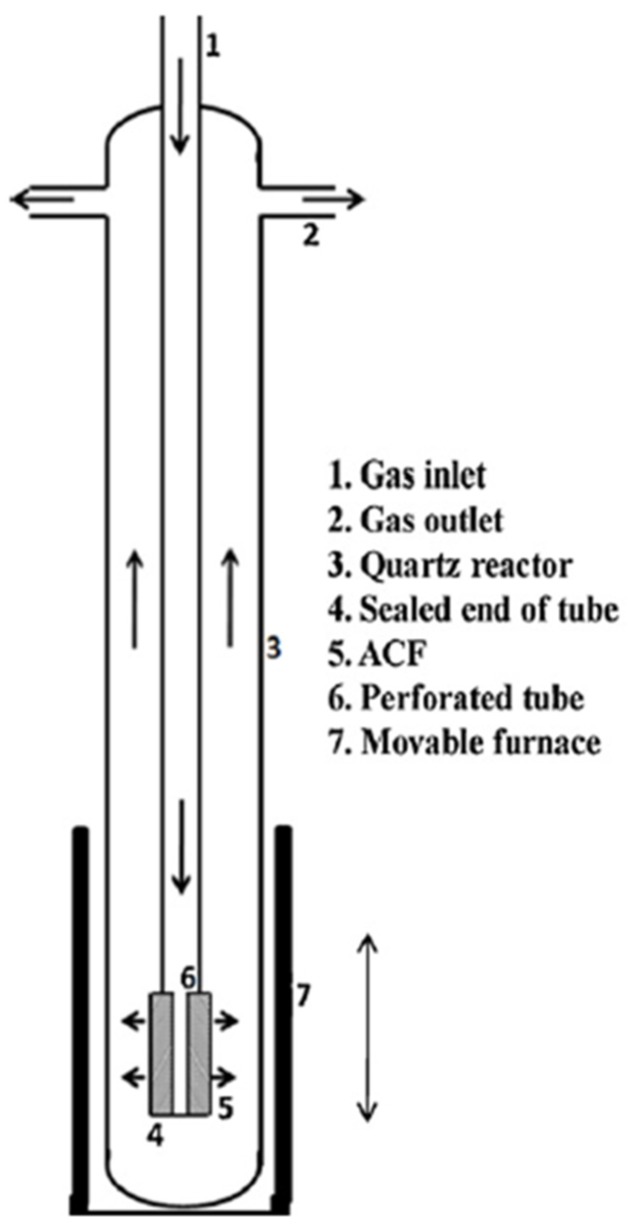
Schematic of activated carbon fiber (ACF) packed CVD reactor. Reproduced with permission from [129]. Copyright (2012) Elsevier B.V.

In other studies, CNFs were grown on activated carbon fibers (ACFs) in a vertical CVD reactor (Figure 13) [129]. The advantages of using a vertical reactor are less of a footprint and that the furnace can be used as a vertical configuration just after CNFs’ growth. This allows the reactor to cool down very quickly and immediate reuse of the reactor in the subsequent CNF production. Another advantage is that the downward flow of the gas in the tube and also radial flow outward through the ACF-cloth wrapped over the perforated section of the vertical tube makes a uniform flow through the ACF and therefore a uniform distribution of CNFs [129].

### 2.4. Graphene

**Figure 14:**
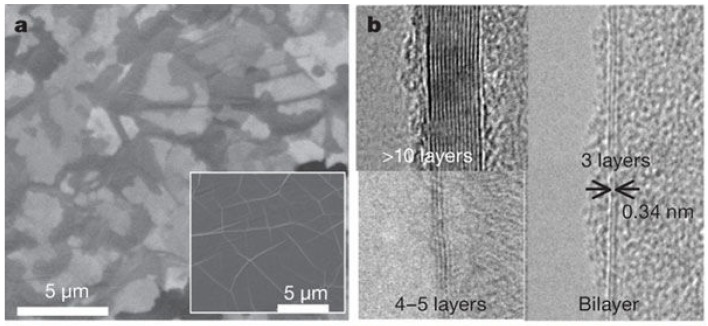
(**a**) Scanning electron microscope (SEM) images of as-grown graphene films on thin (300-nm) nickel layers and thick (1-mm) Ni foils (inset); (**b**) TEM images of graphene films of different thicknesses. Reproduced with permission from [131]. Copyright (2009) Macmillan Publishers Limited.

Graphene is the building block of some other well-known allotropes such as graphite, carbon nanotubes, fullerene, and charcoal. In fact, the graphene allotrope is organized in a two-dimensional honeycomb a hexagonal lattice with carbon atoms located in each vertex [130]. Figure 14 shows scanning electron microscope (SEM) and transmission electron microscope (TEM) images of graphene [131].

**Figure 15:**
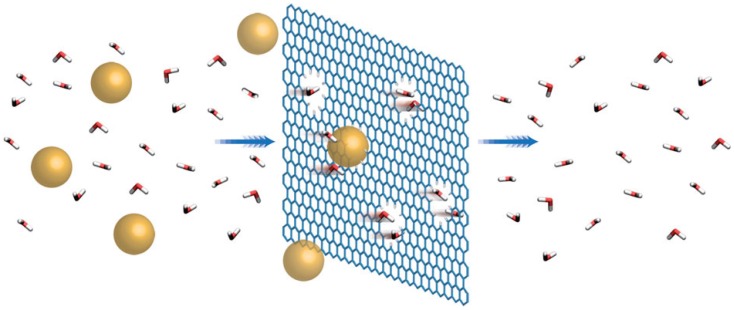
Rejection of pollutants by a graphene sheet. Reproduced with permission from [136]. Copyright (2017) Macmillan Publishers Limited.

A single 2-D sheet of graphene has a hexagonal structure with each atom forming three bonds with each of its neighbors, called σ bonds, oriented towards the closest atoms and formed from three of the valence electrons. The covalent carbon–carbon bonds are almost similar to the bonds in diamond organization and structure, resulting in the similar mechanical and thermal properties of graphene. The fourth valence electron does not participate in covalent bonding. It is in the 2pz state oriented perpendicular to the sheet of graphite and forms a conducting π band. The exclusive electronic property of a carbon nanotube is a direct consequence of π band in the graphene structure. Due to difficulties in separating and isolating graphene sheets, there are not many experimental studies on the mechanical properties of graphene available in literature. Graphene is widely used in many applications due to its exceptional properties such as conductivity (highly conductive to electricity and heat), transparency and strength (100 times stronger than steel by weight). One of the important applications of graphene is the fabrication of water purification and desalination membranes due to its flexibility, mechanical and chemical stability and ability to fabricate one-atom thickness structures [132]. The performance of such membranes has been proved to be superior to the polymeric membranes due to their thinness (one-atom thick) and strength (high-tensile strength) at the same time [133,134]. The thinness of graphene membranes signifies that they have low mass-transport resistance, while high-tensile strength means graphene membranes can withstand high pressure-driven processes such as micro-filtration (MF), nano-filtration (NF) or reverse-osmosis (RO) [133]. Due to its nature, graphene sheets are highly impermeable to several species; hence, the incorporation of graphene sheets into separation membranes will prevent pollutants from passing through the membrane [135]. Defect-free graphene sheets (even if they are one-atom thick) are impermeable because of the repulsive force formed by the dense and delocalized π orbital cloud. The π orbital cloud is believed to fill the gap within its aromatic rings and will have the effect of blocking the smallest molecules such as helium or hydrogen from passing through even under high pressure [136,137]. Figure 15 represents schematic of the rejection of pollutants by a graphene sheet [136].

Ever since they were first introduced into separation and water-purification membranes, graphene membranes have been studied experimentally and they demonstrate high selectivity, flux and fouling resistance. Studies have confirmed that graphene also has the capacity to adsorb CO_2_ [138]. Furthermore, graphene is highly tunable as it can be easily functionalized with so many compounds that will further improve its performance.

#### Synthesis of Graphene via CVD

Using Scotch tape, graphene was first exfoliated mechanically from graphite [139,140]. In 2004, the first attempt to isolate a single graphene layer was reported by Novoselov et al. [140]. In this technique, cleaved graphite crystal gently rubbed or pressed on an oxidized silicon give fresh wafer graphene flakes with the correct thickness of oxide; single atomic layers are visible under an optical microscope due to thin-film interference effects. In this technique Scotch tape, associated with the optical identification on 285 nm SiO_2_ substrates, gave virtually anybody access to such a breakthrough research subject without the need for important resources to acquire and process the material.

Later, further attempts were undertaken to improve the quality and yield of exfoliation techniques. One method is stamping which utilizes silicon pillars by electrostatic voltage-assisted exfoliation to transfer graphene flakes and control separation of the graphene sheet from bulk crystals [141,142]. Another common technique reported in literature is the dispersion of graphene from solution. In this method, graphite flakes are sonicated in a solution and then dispersed on to a wafer. To locate the single graphene sheet, the AFM is used, resulting in a time-consuming process relative to other optical detection schemes. To disperse graphene in solution, very long sonication is needed to break the graphite down and this typically results in small flakes [143]. The disadvantage of this technique is the difficulties in dispersing graphene from the solution and separating the layers without breaking them. Graphene can also be synthesized from the petroleum pitch-derived carbon material through a simple process based on exfoliation with organic solvents [144].

The direct growth of graphene is a potential technique for the mass production of graphene sheets. In this process, SiC wafer is heated and this results in the partial graphitization of the graphene upper layer [145]. With this technique, controlling the number of layers as well as the grain sizes is challenging [146]. To isolate single graphene, lithography is required to pattern electrostatic gates on top of the graphene.

**Figure 16:**
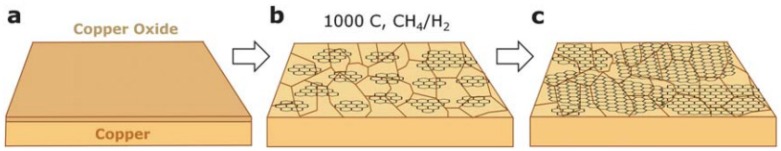
The three main steps of growing graphene on copper by CVD: (**a**) copper foil with native oxide; (**b**) the exposure of the copper foil to CH_4_/H_2_ atmosphere at 1000 °C leading to the nucleation of graphene islands; (**c**) enlargement of the graphene flakes with different lattice orientations. Reproduced with permission from [151]. Copyright (2011) Royal Society of Chemistry.

Nowadays, graphene are synthesized by chemical vapor deposition on the surface of some metals (catalyst) from a carbon-containing gas or through the surface separation of carbon, which is dissolved in the bulk of some metals. The CVD process for the synthesis of graphene has the advantage that relatively high-quality graphene can be produced, potentially on a large scale. The CVD process is reasonably straightforward, although some specialist equipment is necessary. The disadvantages of CVD is that the gaseous by-products of the process are usually very toxic. However, these toxic by-products are usually removed from the reaction chamber by gas flow. In the CVD process, gas mixture (H_2_ and CH_4_) is heated up to 1000 °C before being deposited on the surface of a nickel metal [147]. This forms some sort of concentration gradient between the metal surface and bulk, forcing carbon atoms to diffuse into the surface of the nickel metal before forming graphite when saturation occurs [148,149]. CVD at ambient pressure resulted in the formation of 1–12 layers of graphene on top of polycrystalline nickel films, while decomposition of ethylene on top of pre-annealed platinum (111) has been found to form a single layer of epitaxial graphite [150]. The optimum metal surface for forming a monolayer in the CVD process was found to be copper due to the low solubility of carbon in copper (0.001 atom % at 1000 °C, compared to nickel which has 1.3% at the same temperature) [148]. Figure 16 shows a schematic illustration of the synthesis of graphene on top of copper using CVD [151]. The final step in the graphene synthesis is the etching process, which is intended to detach graphene in order to remove layers of the metal catalyst [152]. The possibility of large-scale production of high-quality graphene films was reported by Li et al. [130] when graphene films were synthesized on the surface of copper that was a centimeter in scale due to the flexibility of copper foils. Nickel, on the other hand, was very rigid and that reduced the graphene films’ production ability to be scaled up [152]. The exceptional flexibility of copper metal foils when used as a substrate has facilitated the roll-to-roll transfer method which has enabled scientists to synthesize 30-inch long graphene sheets [153,154,155].

Besides nickel and copper, other metals can be also used for catalyzing CVD graphene growth such as Pt [156], Co [157], Ir [158,159], and Ru [160,161], but this review focuses more on Cu and Ni, as they are now the most promising candidates for the mass production of graphene.

Based on the type of catalyst, whether Ni or Cu, two fundamental mechanisms happen in graphene formation [130]. If the catalyst is polycrystalline Ni, the precursor decomposes at the surface and carbon dissolves in the metal. Later, when the substrate is cooled and the solubility of the C in Ni decreases, the graphene layers are segregated and then grow on the Ni surface [162]. Therefore, cooling control is an essential step for regulating the number of graphene layers or reaching monolayer graphene [150]. On the other hand, when a Cu catalyst is used, the carbon does not dissolve in the metal due to the low solubility of C in Cu at elevated temperatures. Therefore, the graphene layers are formed directly on the surface of the metal without any need to control the cooling temperature on the metal substrate. So, CVD with copper catalyst is considered to be surface-mediated and self-limiting [162]. Also, when a Cu catalyst is used, monolayer graphene is formed with no further propagation, due to blockage on the surface of the catalytic Cu.

Using different catalyst morphologies requires different mechanisms for the growth of graphene on the surface. For instance, when graphene is grown on surface of Ni(111), a monolayer graphene on Ni(111) single crystal, and multilayer graphene on Ni films may be obtained under the same experimental parameters [163]. It has been reported that graphene growth on Ni(111) is strongly initiated by the Ni(111) lattice due to strong Ni–C bonding and, therefore, forms monolayer graphene. On the other hand, the multilayer graphene flakes formed on polycrystalline Ni films are usually loaded with deviations of the Bernal stacking type and show small rotations among the carbon layers. CVD graphene formed on polycrystalline Ni produces a higher percentage of multilayer graphene because of the grain boundaries in Ni that can serve as nucleation sites for multilayer growth [163].

Another approach in CVD is to use lower-temperature CVD below 600 °C in order to form multilayer graphene by carbon segregation from the bulk. The optimum temperature for graphene growth is reported to be around 550 °C and any reaction above this temperature causes carbon diffusion into the bulk and, therefore, limits the surface growth rate. Conversely, when the temperature is below 500 °C, a competing surface carbide phase impedes graphene formation [164].

In addition to methods using the gas precursor to produce graphene, a wide range of carbon feedstocks has been reported in literature, such as poly(methylmethacrylate) (PMMA), SU8-2002 photoresist, benzene, ethanol, and other carbon sources [165,166]. When SU8-2002 photoresist is used on Ni foil with annealing at 1000 °C in an ambient mixture of He and H_2_ gas, high-quality graphene is synthesized [165].

Another approach is reported by Liu et al. [167] using a Cu–Ni binary alloy. When atomic percentage of Ni in the Cu–Ni alloy is increased, it was observed that a thicker and more uniform graphene layer was formed [167,168,169].

**Figure 17:**
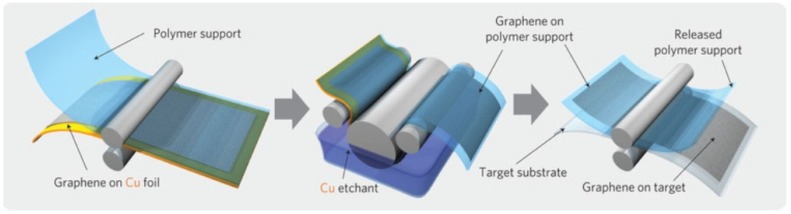
Schematic of the roll-to-roll transfer process illustrating the steps of adhesion of the polymer support, copper etching (rinsing) and dry transfer-printing on a target substrate. Reproduced with permission from [170]. Copyright (2010) Macmillan Publishers Limited.

Graphene nanoribbons (GNRs) are synthesized by unzipping CNTs and are characterized as narrow and elongated graphene stripes (Figure 17) [170]. In this method, controlling the parameters and morphology of graphene sheet is easier compared to other methods. By choosing CNTs with well-defined suitable size and chirality, the characteristics of graphene sheets can be chosen. There are several options for producing GNRs such as oxidative splitting [138], plasma etching [139,140], reductive splitting [141], sonochemical reactions [142], and sputter-etching [143]. Nanoribbons are prepared employing solution-based oxidative processes using KMnO_4_ solution in which it is easily soluble in water due to the presence of a large amount of oxygen species similar to graphene oxide (GO) [139].

Thermal exfoliation based on the reduction of GO has also gained a lot of attention in recent years [171]. The main reason for this is the high accessibility of these methods, with minimum need of sophisticated equipment and high temperatures or very low pressures/vacuum. This technique is a candidate for inexpensive mass production.

Graphene can also be synthesized via ultra-high vacuum plasma-enhanced chemical vapor deposition (UHV-PECVD) and Joule-heating-induced chemical vapor deposition. Adcock [172] proposed a method for fabricating graphene using UHV-PECVD. The new UHV-PECVD system can produce high-quality films by tuning the graphene growth process. It was observed that a nanoscale, crystalline form of graphitic carbon film was deposited. The temperature and growth time were found to be the significant factors that affect the deposition process. Higher plasma power and higher temperature was recommended for more growth.

Lee et al. [173] reported the large-scale synthesis of graphene films in a cold-wall reactor by Joule-heating-induced chemical vapor deposition method. In this method, catalytic metal layers on the SiO_2_/Si substrates are self-heated to high growth temperature (900 °C to 1000 °C) by high-current Joule heating. The metal film is directly connected to the high-current electrodes and is locally heated, which provides several advantages over the hot-wall CVD system. The system was able to produce high-quality graphene films with electrical and structural characteristics comparable to those grown by hot-wall CVD systems.

Another low-cost, effective and simple method for the production of high-quality graphene is radio frequency plasma-enhanced chemical vapor deposition (RF-PECVD). Qi et al. [174] synthesized graphene via RF-PECVD on SiO_2_/Si substrate covered with Ni thin film at relatively low temperatures (650 °C). Methane gas is introduced into a PECVD chamber during deposition and the carbon atom forms single-layer or few-layer graphene on Ni film. After deposition, Ni is removed by wet etching, and the single continuous graphene film obtained can easily be transferred to other substrates.

### 2.5. Carbide-Derived Carbon (CDC)

Carbide-derived carbon (CDC), which is also referred to as “tunable nano-porous carbon”, is the name used to refer to carbon materials that have been derived from two sources, which are:

1. Carbide precursors (such as silicon carbide (SiC) or Titanium carbide (TiC));
2. Ternary carbides, which are also known as MAX phase (such as: Ti_2_AlC or Ti_3_SiC_2_, etc.) [175].

Moreover, CDCs can be derived from carbonitrides such as Si-N-C or from polymer-derived ceramics such as Ti-C or Si-O-C. CDCs can be found in both crystalline and amorphous structures in addition to their existence in both fully dense and highly porous structures [176]. Furthermore, CDCs can be found in both sp^2^ to sp^3^-bonded compounds [177]. Interestingly, CDCs are the origin of some of the carbon-based species such as:

- Carbon nanotubes (CNTs);
- Graphite;
- Graphene;
- Nano-crystalline diamond;
- Onion-like carbon;
- Amorphous carbon;
- Micro-porous carbon (pore size less than 2 nm);
- Meso-porous carbon (pore size between 2 and 50 nm) [175].

The largest specific surface area in the above carbon materials has been found to be microporous carbon, which has an average area of around 3000 square meters per gram [178]. Depending on the synthesis conditions and type of precursor used, both micro and mesoporous carbon can be synthesized with controllable pore size and pore-size distributions. The pore-size control can be as low as sub angstrom accuracy [179]. This outstanding control over pore size has enabled CDCs to be an excellent material for storage and selective sorption of gas and liquids (such as: CO_2_, methane, hydrogen, etc.); moreover, the electrochemical stability and electric conductivity of CDCs have been utilized to fabricate capacitive water desalination membranes and electrical energy storage from CDC-based materials [180].

#### Synthesis of CDC via CVD

Various physical and chemical (CVD) techniques have been used to synthesize CDCs. Microporous CDC is formed from the etching of metals and metalloids from metal carbides. Metals are usually etched with high-temperature halogenation reactions in a CVD unit to form metal halides and porous carbon. Halogens used in the process are Cl_2_, Br_2_, F_2_, and I_2_ along with their compounds such as HF, CCl_4_, and many others can be used to produce CDCs. The porosity of the CDC is dependent on the halogen which is chosen for removal of metals and metalloids [181]. CDCs are also synthesized using hydrothermal leaching and the reaction of certain metal carbides with inorganic salts. The three most commonly used methods are chlorine treatment, vacuum decomposition, and hydrothermal etching [175]. Dry chlorine treatment is the most commonly used technique in which chlorine is used to etch the metal or metalloid atoms from the lattice of the carbide precursor. The term “chlorine treatment” is more commonly used over chlorination since the metal chloride which has been chlorinated is the unwanted byproduct while the carbon element itself is still unreacted. Chlorine treatment is widely used for the commercial production of CDCs [175]. The general equation depicting the reaction of a metal chloride with chlorine gas is shown below:

MC(solid) + 2 Cl_2_(gas) → MCl_4_(gas) + C(solid)

**Figure 18:**
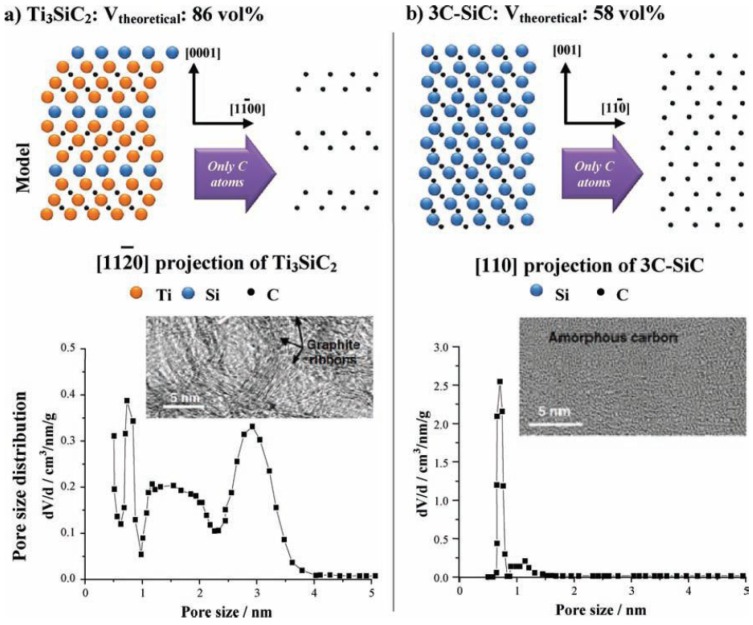
Schematic of the atomic structure of Ti_3_SiC_2_ (**a**) and 3C-SiC (**b**) and the corresponding carbide-derived carbon (CDC) structures after halogenation. Reproduced with permission from [175]. Copyright (2011) Wiley Publishers & (2006) CRC Taylor & Francis.

The reaction above can be analyzed as a process with the selective removal of metal atoms out of a metal carbide matrix. With this process, a large amount of porosity is produced under the conservation of the original shape of the carbide precursor and control over the resulting pore sizes. Typically, the bulk porosity is greater than 50% and specific surface area reaches up to 3200 m^2^/g [175]. These factors depend mainly on the structure of the carbide precursor used. Figure 18 illustrates different distribution of carbon atoms in the carbide for the ternary carbide Ti_3_SiC_2_ and the binary 3C-SiC where the crystal lattice leads to different total porosities [175].

The pore-size distribution (PSD) is essential to the selection of carbide precursor. As an example, ternary carbides like Ti_3_SiC_2_ have broader PSD with multiple maxima whereas binary carbides like SiC show a monomodal and narrow PSD (Figure 18) [175]. In addition, the temperature of chlorination has a major effect on the PSD. As temperature increases, the pore size also increases due to the beginning of graphitization as a result of the self-organization of the highly mobile carbon atoms. These parameters are important for pores in the range of micropore and small mesopore. For the synthesis of larger pores or even the insertion of hierarchical porosity, templating techniques are used.

Moreover, vacuum decomposition of SiC wafers has also been used to prepare epitaxial graphene with homogeneous areas [175]. This method involves the extraction of the atoms of metal/metalloid elements under vacuum at high temperatures (>1200 °C). The high melting point of carbon relative to carbide metals is the key that drives this method; at high temperature, carbide metals melt and evaporate while leaving the carbon element in the physical state.

Nanodiamond and nanoporous carbon films are prepared from SiC-CDC that has been pre-synthesized by the hydrothermal etching technique. This technique takes place at high temperatures (300–1000 °C) and the subsequent reactions between water and metal carbides take place:

^x^/_2_•MC + x•H_2_O → M_x/2_O_x_ + ^x^/_2_•CH_4_

MC + (x + 1)•H_2_O → MO_x_ + CO + (x + 1)•H_2_

MC + (x + 2)•H_2_O → MO_x_ + CO_2_ + (x + 2)•H_2_

MC + x•H_2_O → MO_x_ + C + x•H_2_

Only the last reaction gives solid carbon. This technique has been found to form carbon from various sources such as TiC, SiC, NbC, TaC and WC [182].

These days, more attention is focused on the carbon formed, with numerous binary carbide powders including Al_4_C_3_ [183], B_4_C [184], Cr_3_C_2_, HfC, Mo_2_C, NbC, SiC [185], TaC, TiC [186], VC and ZrC [187] as precursors for CDCs. In addition, CDC also been synthesized from other carbide forms including bulk, nano-wires [188], and whiskers [189]. Additionally, bulk samples have been partially chlorinated to produce thin-film CDC layers [190,191].

### 2.6. Carbon Onion

**Figure 19:**
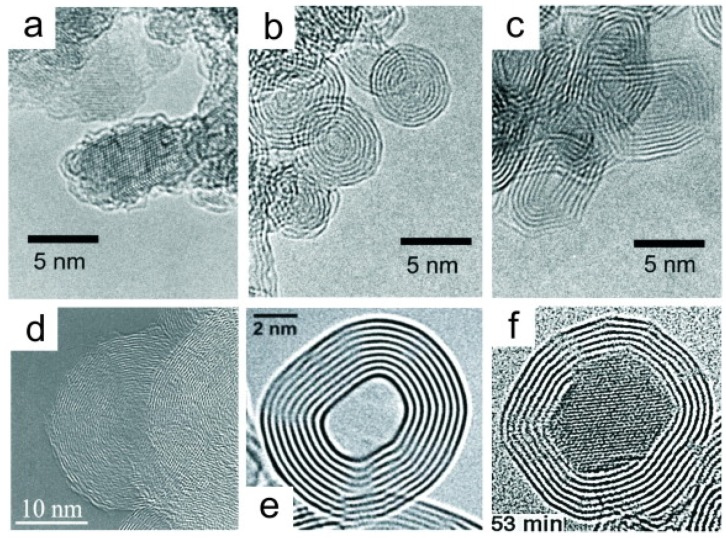
High-resolution transmission electron microscope (HRTEM) images, high resolution of (**a**) NDs [196]; (**b**) spherical ”small” carbon nano-onions (CNOs) [196]; (**c**) polyhedral CNOs [196]; (**d**) spherical “big” CNOs [197]; (**e**) spherical hollow-core CNOs [198]; and (**f**) metal-core CNOs [199]. Reproduced with permission from [193,196,197,198,199]. Copyright (2001 & 2002) AIP Publishing LLC, (2008) John Wiley and Sons, and (1998 & 2017) Elsevier B.V.

Carbon onions were discovered a long time before CNTs and fullerene; however, they stayed in the shadows of the more well investigated and popular form of carbon-based materials (CNTs and fullerene) [192]. Nowadays, carbon onion has been well studied and identified as a carbon-based nanomaterial that can be used in various applications such as energy storage and electronics [192]. Carbon onions can be defined as carbon shells which are spherically-closed. The reason they are called carbon onions is due to their structure (concentric layered shape) which looks like an onion, as shown in Figure 19 [193]. Carbon onions are sometimes referred to as onion-like carbon (OLC) or carbon nano-onions (CNOs). In fact, CNOs can be defined as multi-layered or multi-shelled structures of fullerene. CNOs include all kinds of concentric shells starting from nested fullerenes to small polyhedral nanostructures (<100 nm) [193]. Following the discovery of fullerenes, CNOs were discovered in 1980 by Sumio Iijima [32]. He accidentally discovered CNOs when he was inspecting a carbon black sample using a transmission electron microscope [194]. CNOs were not produced in bulk; however, they were produced as a byproduct of the synthesis of carbon black [193]. Twelve years after that, Ugarte reported a mechanism to synthesize spherical CNOs [195]. By directing an electron beam on a sample of carbon which is in the amorphous state, Ugarte successfully synthesized CNOs in situ [195]. Normally, amorphous carbon graphitizes and starts to curl when exposed to electron beams; after some time, the graphitic carbon was found to close on itself and start forming onion-like structures [195]. The reason behind the curving and closure of graphitic structures is thought to be due to the reduction of the surface energy of the recently formed graphitic edge planes, which is around 30 times that of the basal plane [194].

#### Synthesis of Carbon Onion via CVD

**Figure 20:**
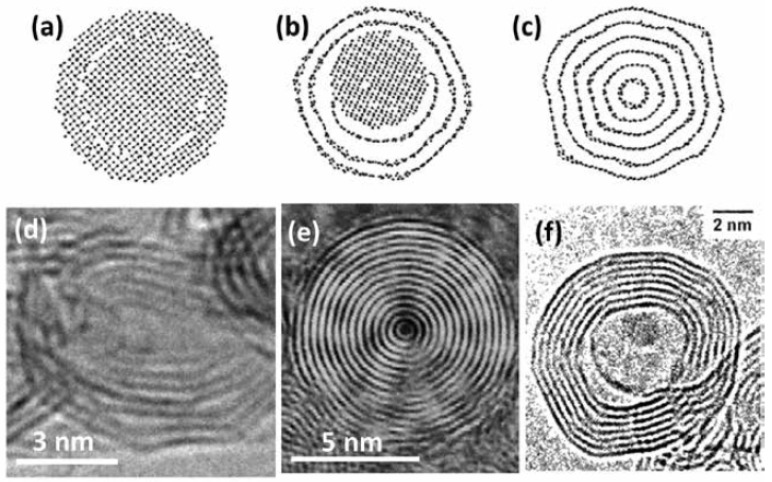
Molecular dynamics simulation of (**a**) pristine nanodiamond; (**b**) nanodiamond annealed at 1400 °C; (**c**) nanodiamond annealed at 2000 °C [207]; and carbon onions synthesized via (**d**) annealing of nanodiamond at 2000 °C [208] (**e**) arc discharge between two carbon electrodes in water [203]; and (**f**) electron-beam irradiation. Reproduced with permission from [192,203,204,205,206]. Copyright (2013) The Electrochemical Society, (2011 & 2012) Elsevier B.V., (2004) AIP Publishing LLC.

Since they were first discovered, there are several methods used to synthesize CNOs; however, large-scale production of CNOs (in the gram scale) was achieved by in 1994 by Vladimir Kuznetsov and his coworkers [200,201]. They used vacuum annealing of a nanodiamond precursor. Other research groups have also used the same synthesis technique (annealing) employing inert gases to convert nanodiamond (synthesized in huge amounts) into CNOs [202]. This technique can be utilized in industry to synthesize CNOs commercially as the yield is almost 100% and can be scaled up easily [192]. Normally, the CNOs produced rarely have a spherical shape; however, they perform well in their applications [192]. Figure 20a–c depicts the transformation of nanodiamond into CNOs using a simulation of molecular dynamics [192,203,204,205,206]. Figure 20a shows 2 nm nanodiamond structure while Figure 20b depicts the same nanodiamond after annealing at 1400 °C; this high-temperature annealing has forced the outer layers of nanodiamond to be transformed into graphitic carbon [192]. Unfortunately, this temperature is not high enough to transform the whole nanodiamond [192]. Figure 20c shows the transformation of the whole nanodiamond into CNOs at 2000 °C [192]. As shown in Figure 20d, the high-temperature annealing initiates CNO polygonization and causes the nanoscale structure to become ordered [207]. CNO is highly reliant on its precursor (nanodiamond). Normally, the nanodiamond has a diameter of around 5 nm while CNOs have 5–10 nm [192,208].

Arc discharge between two graphitic electrodes in water is another technique used to synthesize CNOs which have marginally different structures than those produced through annealing [206]. This method involves applying direct current (17 volts and 30 amperes) between the two graphitic electrodes immersed in water. Due to the high heat generated, evaporation of carbon (at the arc) starts to occur, which is then condensed into spherical CNO (Figure 20e) and floats on the surface of the water [197]. This method enables synthesis of CNOs at room temperature and pressure and avoids using catalysts; unfortunately, the yield is low [197,203,206].

Metal nanoparticles have been used to synthesize hollow carbon onions by evaporating carbon and metal using the arc discharge technique. This method will produce some metal particles encapsulated by layers of graphitic carbon [192]. Once they are exposed to a transmission electron microscope beam, particles of metal migrate a few atoms at a time through carbon layers and leave hollow CNO particles, as shown in Figure 20f [209].

**Figure 21:**
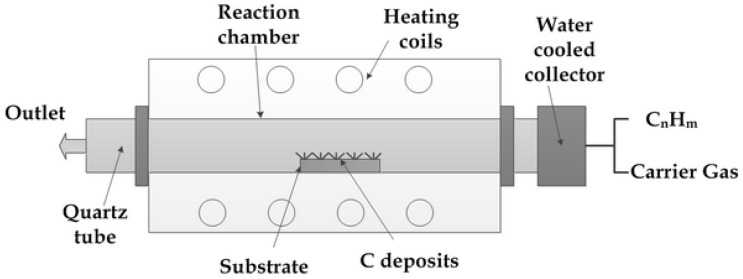
Chemical vapor deposition of CNO. Reproduced with permission from [204]. Copyright (2011) The author(s) and IN TECH.

Moreover, CNOs can also be synthesized using the CVD method with iron as the catalyst on a sodium chloride support. The decomposition of acetylene gas at 400 °C produces larger diameter CNOs (50 nm) when compared to other CNO preparation methods [192]. Figure 21 shows a schematic diagram of the synthesis of CNO using CVD [204]. Furthermore, carbon ion implantation is a successful technique for synthesizing CNOs with an ability to tune the CNO diameter from 3 nm to 30 nm by changing the operating conditions, such as implantation-dose density and temperature [192]. This method was introduced in 1998 by Cabioc’h et al. [210]. Larger diameter CNOs (40 nm) have also been synthesized by solid-state carbonization of phenolic resin precursor [211]. The reaction involves the catalyst ferric nitrate and precursor phenol-formaldehyde resins and takes place at temperatures around 1000 °C ,which results in the evaporation of nanodiamond followed by condensation (on a silicon substrate) of carbon in the form of CNOs [211].

A.V. Kabashin and co-workers [212] synthesized CNO with a novel pulsed laser-assisted method. Later, this method was utilized to grow CNTs [205,213,214,215]. Y.S. Zhou and co-workers [216] have also reported on their work with the laser-assisted nanofabrication of CNOs. In this method, exposing a material’s surface to a laser have consequences, such as a narrow heating zone, melting, photochemical reactions, decomposition, etc. The irradiation of the surface of the material by photon beams at resonant excitation breaks these bonds and produces localized chemical reactions.

### 2.7. MXene

The presence of highly fouled saline and wastewater that need to be desalinated/purified, in addition to highly pressurized processes, have necessitated the search for novel materials which can withstand the harsh operating conditions present in the field of water purification and desalination. Novel materials such as CNTs and graphene-based membranes have demonstrated excellent mechanical properties, high water flux in addition to easy surface functionalization. For example, GO nanosheets have enabled the fabrication of ultrathin atom-thick sieving membranes as a result of their flexibility and outstanding dispersion in aqueous solutions; however, the stability and selectivity of wetted GO membranes in crossflow filtration/desalination is still a hot topic for researchers as the high solubility of GO nanosheets cause disintegration of the membrane when exposed to filtration-operating conditions. This has opened the door for scientists to introduce a new material that is highly permeable and stable (both at the chemical and mechanical level). This material is referred to as MXene. MXenes are a member of MAX phase groups which are 2D inorganic compounds [217]. The MAX Phases can be defined as layered, hexagonal carbides and nitrides (+60 members) which have the general formula of M*_n_*_+1_AX*_n_*, (MAX) where M is an early transition metal (such as: Ti, V, Cr, Nb, etc.), A is an A-group (such as: Al, Si, Sn, In, etc.) element, X is either carbon and/or nitrogen and *n* is 1 to 3 [218]. MXenes, which are part of the MAX phase, consist of few atoms-thick layers of transition metal-carbide or carbon-nitride [172].

#### Synthesis of MXene via CVD

**Figure 22:**
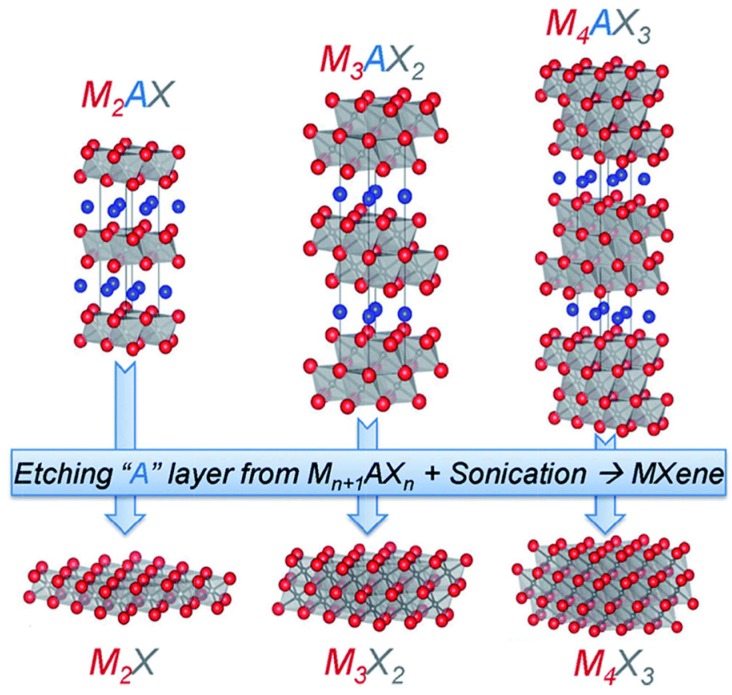
Structure and synthesis of MXenes from MAX phase. Reproduced with permission from [219]. Copyright (2015) Royal Society of Chemistry.

The synthesis of MXene involves removing the A of MAX compounds by selective etching, as shown in Figure 22 [219].

**Figure 23:**
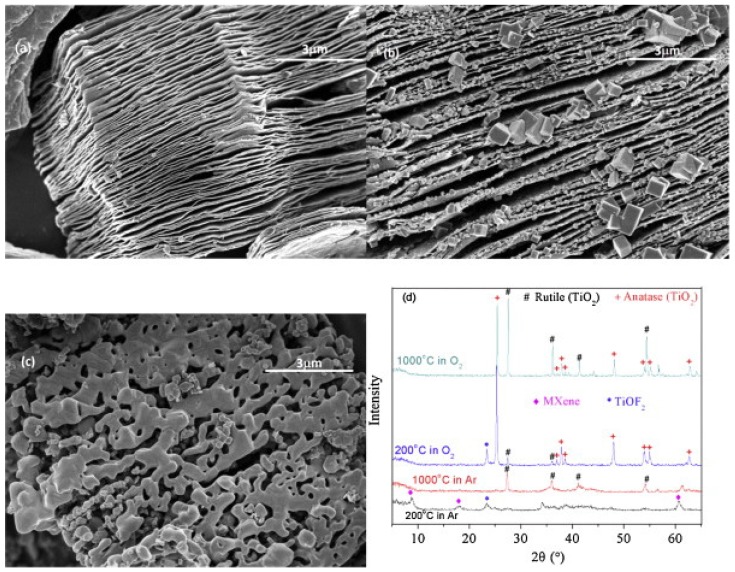
(**a**) SEM image of MXene after thermal analysis at 1000 °C in Ar; (**b**) SEM image of MXene after thermal analysis at 200 °C in O_2_; (**c**) SEM image of MXene after thermal analysis at 1000 °C in O_2_; and (**d**) X-ray diffraction (XRD) patterns of MXene after thermal analysis in O_2_ or in Ar. Reproduced with permission from [220]. Copyright (2015) Elsevier B.V.

Figure 23 shows the SEM and X-ray diffraction (XRD) results of MXene samples after high temperature processing in a thermal analyzer [220]. Figure 23a is the SEM micrograph of a MXene sample processed at 1000 °C in Ar atmosphere. The sample still kept the loose quasi-2D structure of MXene. Figure 22b,c are the SEM micrographs of sample processed in O_2_ atmosphere at 200 °C or 1000 °C, respectively. From Figure 22b, at 200 °C, many equiaxial crystals were formed on the surface or edge of the 2D structure. A few crystals are large (~1 μm) and most crystals are small (~100 nm). However, the quasi-2D structure of MXene was kept.

Due to their exceptional properties, MXenes have been a hot topic for scientists in the field of energy storage for fabricating lithium-ion batteries. These interesting compounds have been referred to as MXenes as they are synthesized by etching the A layer from the MAX phase compounds, which turns MAX into MX [221]. Moreover, the suffix “ene” was added to stress the similarity (in structure) of these compounds with graphene (both are 2D) [221].

The bandgap of MXenes can be tuned easily by changing the surface termination of MXene; for instance, bare MXene is a metallic conductor while F or OH^–^ terminated ones are semiconductors with a small band gap [222]. The conductivity of multilayer MXenes has been found to be similar to that of multilayer graphene (both are electronically conductive) [223]. Moreover, MXenes have hydrophilic properties unlike graphene which facilitate the easy dispersion of MXenes in aqueous solutions. MXenes have been found to be intercalated with numerous inorganic molecules and organic salts, which enables the preparation of different intercalation compounds in addition to discovery of new applications for these materials [223]. The exceptional properties of MXenes have enabled them to be used in sensors, electronic devices, energy-storage materials and composite reinforcement [224].

When comparing MXenes to the available layered materials such as graphene, MXenes show superior qualities in terms of stability and strength. For example, graphene layers are connected by van der Waals bonds which are weak; however, layers of MXenes are too hard to be split in case of applied mechanical or shear stress [217]. The production of MXene flakes requires chemical etching followed by intercalation and sonication. The strength and stability of MXene have the potential to replace other carbon nano-based materials in membrane fabrication [217].

**Figure 24:**
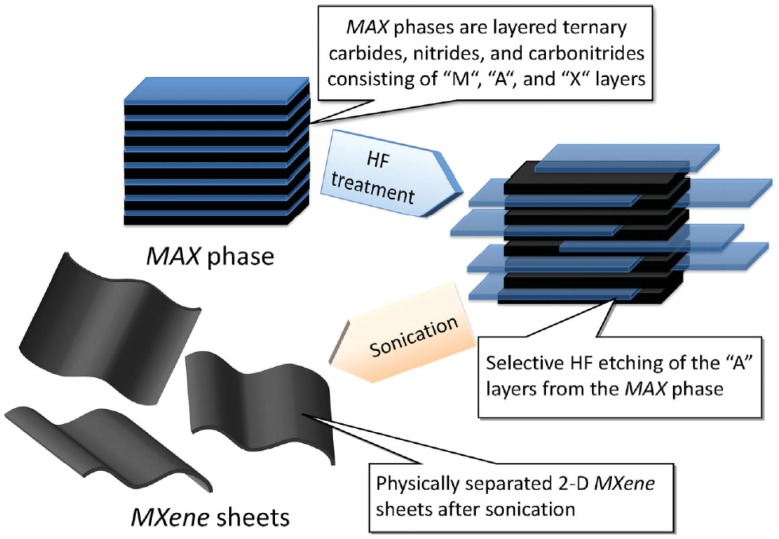
The preparation of MXenes from MAX phases. Reproduced with permission from [226]. Copyright (2012) American Chemical Society.

Depending on the chemical-etching technique and the form of post-treatment, normally MXenes are attached to hydroxyl, oxygen and or fluoride groups [225]. Delamination of the two-dimensional MXenes can be undertaken by sonication which will produce both single-layer and few-layered flakes [225]. Once dried, parts of the hydroxyl groups in the MXenes will be transformed into an oxygen end through the elimination of water molecules. The majority of current studies assume full termination by oxygen, hydroxyl and fluoride groups. Moreover, the interlayer interactions are affected by the presence of water molecules (hydrogen-bond formations) on the surface of MXene layers [225]. Normally, the multilayer MXenes produced act differently to the single-delaminated or few-layer Ti_3_C_2_T_x_ MXenes. Of the various MXenes available, Ti_3_C_2_ is the most widely covered MXene in literature and can be delaminated in large quantities, which opens the potential for commercial production [226]. A schematic of the exfoliation process is shown in Figure 24 [226].

## 3. Current Challenges and Future Outlook

In recent years, the synthesis and applications of various carbon nanomaterials have been extensively explored. Although a number of methods are reported in the published literature for the production of these nanomaterials, large-scale economical production of these nanomaterials is still a challenge for researchers.

Secondly, most of these materials are employed in various applications on a lab scale only. One of the main hurdles that limit the application of these nanomaterials in large-scale operation is their high cost. The current price of these nanomaterials does not suggest their application on a large scale. It is expected that due to the increase in commercial production of carbon nanomaterials, their price will be greatly reduced in future. Furthermore, the potential hazardous effects of most of the carbon nanomaterials on human health and the environment also need to be studied in detail.

## 4. Conclusions

In this review paper, the CVD technique for the synthesis of various carbon nanomaterials such as fullerene, CNTs, CNFs, graphene, CDC, CNO and MXenes was discussed. These carbon nanomaterials have been widely used in many applications due to their unique properties such as strength, availability, hydrophilicity, in addition to their ease of fabrication and anti-bacterial behavior. CVD is the most promising technique for the production of 1D, 2D, and 3D carbon-based nanomaterials. However, large-scale production of high-quality carbon nanomaterials is a key future challenge to enabling industries to use them as a raw material for currentl applications. Further research is required to explore the potential toxic effect of these materials and discover economical methods for their commercial production.

## Figures and Tables

**Table 1:** Catalytic production of CNTs by CVD.

Catalyst	Carbon Source/Gas Phase	Temperature (°C)	Product	Reference
Co, Ni, Fe/MgO	CH_4_/H_2_	1000	SWCNTs	[53]
Fe/Al_2_O_3_	C_2_H_4_/N_2_, H_2_	650	MWCNTs	[83]
Fe/Al_2_O_3_	C_2_H_4_/N_2_, H_2_	500–700	MWCNTs	[84]
Ni-Cu/Al_2_O_3_	C_2_H_4_/N_2_, H_2_	850	MWCNTs	[85]
Fe/SiO_2_/Al_2_O_3_	Propylene/N_2_	-	MWCNTs	[86]
Ni/SiO_2_	CH_4_/Ar	760	SWCNTs	[87]
Fe/Al_2_O_3_	Ethylene/N_2_, H_2_	550	CNTs	[88]
Fe/silica	Acetylene/N_2_, H_2_	700	CNTs	[89]
Fe/Al_2_O_3_, SiO_2_, TiO_2_ or ZrO_2_	CH_4_/H_2_	650–800	MWCNTs	[90]
LaCoO_3_	C_2_H_2_/N_2_, H_2_	675–700	MWCNTs	[91]
Co-Mo/SiO_2_	CO	750	SWCNTs	[92]
LaCoO_3_	C_2_H_2_, CH_4_/N_2_	700	MWCNTs	[93]
Fe_2_O_3_	CH_4_/Ar	1000	SWCNTs	[94]
Ni-Cu-Al	CH_4_/N_2_, H_2_	700–750	CNTs	[95]
Fe	C_6_H_6_/Ar	750	MWCNTs	[96]
Ni/Fe/CO/HZSM-5 Zeaolite	Polypropylene (PP), polyethylene terephthalate (PET), polyethylene (PE), Polyvinyl chloride(PVC), PET/Ar, H_2_	400–900	MWCNTs	[97]
NiO/HZSM-5 Zeolite	polypropylene (PP)/H_2_	500–800	MWCNTs	[98]
Fe	PP,PE, PVC/Ar, H_2_	800	MWCNTs	[99]
Si/SiO_2_	CH_4_/H_2_	900	SWCNTs	[100]
Fe_2_CO/Al_2_O_3_	C_2_H_4_/Ar, H_2_	750	MWCNTs	[101]
Si/SiO_2_/Al_2_O_3_	C_6_H_12_/H_2_	750	MWCNTs	[102]
Ni	C_2_H_2_/H_2_	550	MWCNTs	[103]
Si	C_2_H_2_/H_2_, (Fe(CO)_5_	600–750	MWCNTs	[104]
Si_3_N_4_	C_2_H_2_	800–1000	SWCNTs	[105]
Ba/Ca	C_2_H_2_/H_2_, Ar	700	MWCNTs	[106]
Fe(CO)_5_	CO	800–1200	SWCNTs	[107]
Fe-Mo	CH_4_/Ar	875	DWCNTs	[108]
